# Targeting uPAR by CRISPR/Cas9 System Attenuates Cancer Malignancy and Multidrug Resistance

**DOI:** 10.3389/fonc.2019.00080

**Published:** 2019-02-27

**Authors:** Kun Wang, Zi-Hao Xing, Qi-Wei Jiang, Yang Yang, Jia-Rong Huang, Meng-Ling Yuan, Meng-Ning Wei, Yao Li, Sheng-Te Wang, Kun Liu, Zhi Shi

**Affiliations:** Guangdong Provincial Key Laboratory of Bioengineering Medicine, Department of Cell Biology and Institute of Biomedicine, College of Life Science and Technology, Jinan University, National Engineering Research Center of Genetic Medicine, Guangzhou, China

**Keywords:** cancer, uPAR, CRISPR/Cas9, malignancy, drug resistance

## Abstract

Urokinase plasminogen activator receptor (uPAR), a member of the lymphocyte antigen 6 protein superfamily, is overexpressed in different types of cancers and plays an important role in tumorigenesis and development. In this study, we successfully targeted uPAR by CRISPR/Cas9 system in two human cancer cell lines with two individual sgRNAs. Knockout of uPAR inhibited cell proliferation, migration and invasion. Furthermore, knockout of uPAR decreases resistance to 5-FU, cisplatin, docetaxel, and doxorubicin in these cells. Although there are several limitations in the application of CRISPR/Cas9 system for cancer patients, our study offers valuable evidences for the role of uPAR in cancer malignancy and drug resistance.

## Introduction

Urokinase plasminogen activator (uPA) receptor (uPAR), also known as CD87 and encoded by PLAUR gene, is a member of the lymphocyte antigen 6 protein superfamily (1). uPAR is a glycoprotein consisting of 313 amino acid residues with only the extracellular domain, no transmembrane and intracellular structures, and is attached to the cell membrane via glycosylphosphatidylinositol anchors (1). uPAR binds to and activates uPA to cleaving plasminogen to plasmin, thus triggering the remodeling of extracellular matrix and playing a key role in cell adhesion, migration, proliferation, and survival (2). Besides uPA, uPAR can interact with other proteins, including vitronectin, integrins, and EGFR, etc to regulate multiple signal pathways (2). Compared to normal tissues, uPAR is highly expressed in many human cancers including lung, breast, gastric, colorectal, pancreatic, bladder, and prostate cancers, etc (3). The expression of uPAR in these cancers promotes the proliferation, metastasis, and invasion of cancer cells (3). Therefore, uPAR may be an important biomarker and target for cancers. Indeed, many inhibitors of uPAR have been developed. The inhibitors blocks the interaction of uPAR with uPA, including: small molecules UK1 (4), WX-UK1 (5), WX-671 (6), etc; peptides Mupain-1 (7), AE105 (8), ATF (9), etc; and monoclonal antibody ATN-291 (10). In addition, there are inhibitors that inhibit the interaction of uPAR with integrins, including: peptides P25 (11), a325 (12), H245A (13), etc; and monoclonal antibody ATN-658 (14). However, the poor affinity and bioavailability limit the application of these inhibitors in clinic. Consequently, it is necessary to develop new approaches to target uPAR for treatment cancer and other diseases.

The RNA-guided clustered regularly interspaced short palindromic (CRISPR) in combination with a CRISPR-associated nuclease 9 (Cas9) nuclease system is a novel gene editing technology by delivering the Cas9 complexed with a synthetic guide RNA (gRNA) into a cell to cut the desired genome location, allowing existing genes to be removed and/or new ones added (15). Due to the advantages of faster, cheaper, more accurate, and efficient, CRISPR/Cas9 system has been widely used as a basic biology research tool, development of biotechnology products and potentially to treat diseases (16). In this study, we used CRISPR/Cas9 system targeting uPAR to verify the role of uPAR in cancers.

## Materials and Methods

### Cells and Reagents

The two multidrug resistant cancer cell lines HCT8/T and KB_V200_ were cultured in Dulbecco's modified Eagle's medium (DMEM) with 10% FBS, penicillin (100 U/ml) and streptomycin (100 ng/ml) at 37°C in a humidified atmosphere of 5% CO_2_. Restriction endonuclease BsmBI was from New England Biolabs. Polyetherimide (PEI) was from Ploysciences. Cisplatin was from Shandong Qilu Pharmaceutical. 5-FU, docetaxel, and doxorubicin were from LC Laboratories. Puromycin was from Selleck Chemicals. Methylthiazolyldiphenyl-tetrazolium bromide (MTT) was from ApexBio Technology. Anti-uPAR (D121140) antibody was from Shanghai sangon biotech. Anti-Vinculin antibody (BM1611) was from Wuhan Boster Biotech.

### Vector Generation, Lentivirus Production, and Transduction

LentiCRISPRv2 vector (from Addgene #52961) was digested with BsmBI and ligated with annealed oligonucleotides (uPAR-sg1-F: 5′-CACCGGACCAACGGGGATTGCCGTG-3′, uPAR-sg1-R: 5′-AA-ACCACGGCAATCCCCGTTGGTCC-3′; uPAR-sg2-F: 5′-CACCGGGACCACGATCGTGCGCTTG-3′, uPAR-sg2-R: 5′-AAACCAAGCGCACGATCGTGGTCCC-3′). HEK293T were transfected using PEI at 70% confluency with recombinant vectors and packaging vectors pMD2G and psPAX2. Viral supernatant was harvested 96 h after transfection and stored at −80°C. HCT-8/T and KB_V200_ cells were transducted with viral supernatant containing 10 μg/ml polybrene, and were selected with 100 and 10 μg/ml puromycin respectively to establish the stable cell lines.

### Genomic PCR and Sequencing Analysis

The genomic DNA of cells was extracted with the QuickExtract DNA extraction kit following the manufacturer's protocol and amplified with a pair of primers (Detection 1-F: 5′-GACAACGGACAGACTGGAA-3′, Detection 1-R: 5′-CCGAATCGCTCTAAGTGG-3′) designed for the target region of interest using a Pfu DNA polymerase. Followed by agarose gel electrophoresis and ethidium bromide staining, the purified PCR products were sequencing with an ABI 3131xl Genetic analyzer.

### Western Blot Analysis

Cells were harvested and lysed in RIPA buffer (1% NP-40, 0.5% sodium deoxycholate, 0.1%SDS, 10 ng/ml PMSF, 0.03% aprotinin, 1 μM sodium orthovanadate) at 4°C for 30 min. Lysates were centrifuged for 10 min at 14,000 × g and supernants were stored at −80°C as whole cell extracts. Protein concentration was quantified using with Bradford assay. Proteins were separated on 10% SDS-PAGE gels and transferred to polyvinylidene difluoride membranes. Membranes were blocked with 5% BSA and incubated with the indicated primary antibodies. Corresponding horseradish peroxidase-conjugated secondary antibodies were used against each primary antibody. Proteins were detected using the chemiluminescent detection reagents and films.

### Cell Morphology Assay

Cells were seeded on glass cover slips for 24 h and then fixed in 4% paraformaldehyde for 20 min and permeabilized with 0.1% Triton X-100 for 15 min at room temperature. The coverslips were incubated in the dark with 100 nM rhodamine-phalloidin at room temperature for 30 min. Nuclei were counterstained with 100 nM DAPI. The coverslips were rinsed in PBS and inverted on a drop of anti-fade mounting media on a glass slide. Then, these slides were sealed with neutral balsam and viewed under the confocal microscope.

### Cell Viability Assay

Cells were seeded into a 96-well plate at a density of 5,000 cells/well and treated with various concentrations of agents for 72 h. Then 10 μl MTT was added to each well at a final concentration of 0.5 mg/ml. After incubation for 4 h, formazan crystals were dissolved in 50 μl of DMSO, and absorbance at 570 nm was measured by plate reader. The concentrations required to inhibit growth by 50% (IC_50_) were calculated from survival curves as previously described (17).

### Sphere Formation Assay

Cells were trypsinized, suspended in medium containing 0.3% agar and 10% FBS and seeded at a density of 5 × 10^2^ cells/well in a 12-well plate. The agar–cell mixture was plated onto a bottom layer with 0.5% agar. Then treated cells were incubated in a humidified incubator and fresh medium was added every 3 days. Two weeks later, colonies were analyzed microscopically.

### Cell Migration Assay

Cells were seeded into a 6-well plate, and reached 80–90% confluence, the cell monolayer was wounded using a sterilized 10 μl pipette tip and washed with PBS two times. Cells were allowed to migrate for 12, 24, and 36 h in serum-free medium, and the wounds were observed and captured. The gap lengths were measured from the photomicrographs.

### Cell Invasion Assay

Cell invasion assays were performed with a modified Boyden chamber (Corning) containing matrigel-coated polycarbonate membrane filter (6.5 mm diameter, 8 μm pore size). Cells were plated in the upper chamber and the lower chamber contained medium with 10% FBS, and incubated for 24 h at 37°C in 5% CO_2_. Non-migrated cells were scraped from the upper surface of the membrane, and migrated cells remaining on the bottom surface were photographed and counted.

### Statistical Analysis

The experimental data of this paper are the results of three independent repetitions. The data obtained is presented in the form of an average and a standard deviation. Statistical analysis of data differences using *t*-test method. A *P*-value of <0.05 was set as the criterion for statistical significance.

## Results

### Knockout of uPAR by CRISPR/Cas9 System

**Figure 1:**
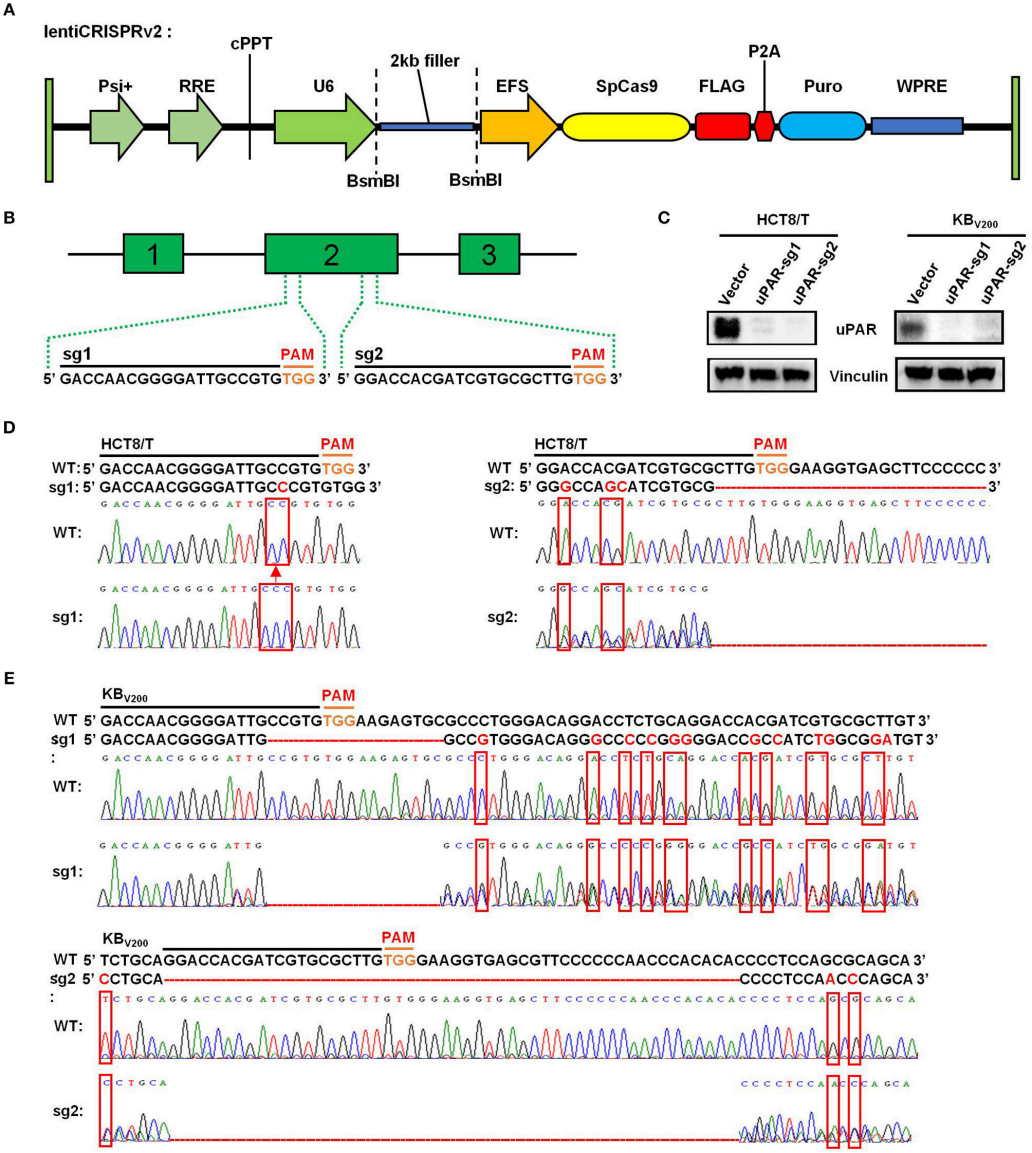
Knockout of uPAR by CRISPR/Cas9 system. **(A)** The map of lentiCRISPRv2 vector. **(B)** The locations and sequences of two sgRNAs of uPAR. **(C)** The protein expression levels of uPAR were examined by Western blot, and vinculin was used as loading control. The genomic DNA of cells was amplified and sequenced by the designed primers. The sequencing comparison and original data of HCT8/T **(D)** and KB_V200_ **(E)** cells are shown.

To target uPAR with CRISPR/Cas9 system, we firstly used lentiCRISPRv2 vector which expresses both hSpCas9 and the chimeric guide RNA (Figure 1A) linked respectively, with two targeting sequences from exon 2 of human uPAR gene (PLAUR) end with a 5′NGG3′ PAM (protospacer adjacent motif) sequence (Figure 1B). Then, the two successfully generated vectors expressed sgRNA1 (sg1) or sgRNA2 (sg2) to target uPAR were identified by sequencing. To establish cell lines stably expressed sgRNA to target uPAR, HCT8/T, and KB_V200_ cells were selected with puromycin after transduction with LentiCRISPRv2 viral supernatant. As shown in Figure 1C, the protein levels of uPAR were undetectable by western blot in both HCT8/T and KB_V200_ cells stably expressed either sg1 or sg2. To further identify the genomic change of targeting uPAR by CRISPR/Cas9 system, the genomic DNA of cells was extracted and amplified using the designed primers by PCR reaction. The sequencing results of PCR productions showed that 1 base was inserted into the target position of HCT8/T uPAR-sg1 cells and 3 base mismatches and a large deletion in the target position of HCT8/T uPAR-sg2 cells (Figure 1D). There were 16 base deletions and 12 base mismatches in the target position of KB_V200_ uPAR-sg1 cells and 51 base deletions and 3 base mismatches in the target position of KB_V200_ uPAR-sg2 cells (Figure 1E). These data suggest that cells with stable knockout of uPAR by CRISPR/Cas9 system were successfully established.

### Knockout of uPAR Alters Cell Morphology

**Figure 2:**
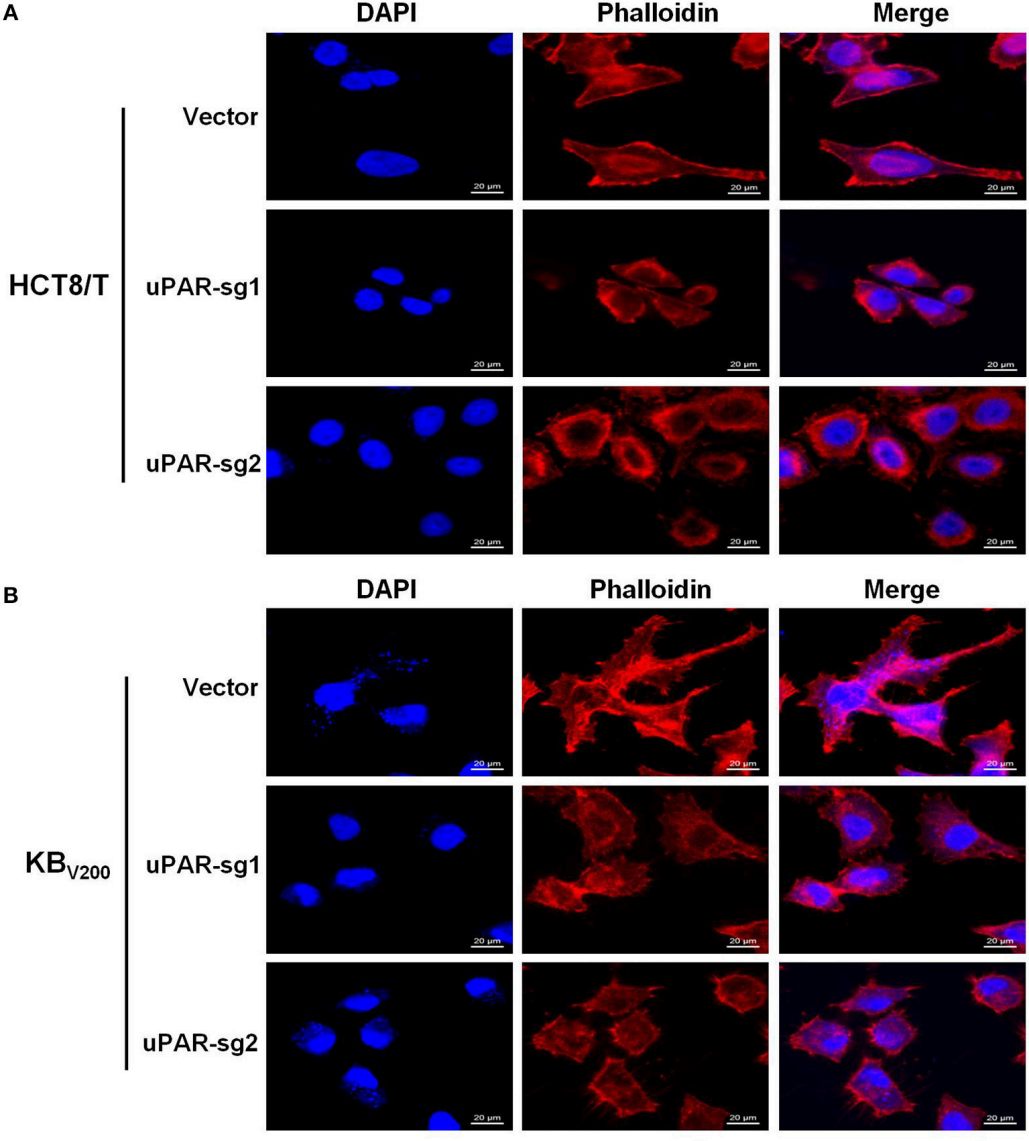
Knockout of uPAR alters cell morphology. The morphology of HCT8/T **(A)** and KB_V200_ **(B)** cells was obtained with confocal microscope.

To explore the effect of knockout of uPAR on cell morphology, we stained cells with Rhodamine-labeled phalloidin and DAPI. The results showed that HCT8/T and KB_V200_ cells with uPAR knockout underwent morphologic changes from spindle-shaped phenotype to round phenotype (Figures 2A,B), indicating that knockout of uPAR alters cell morphology.

### Knockout of uPAR Attenuates Cell Proliferation

**Figure 3:**
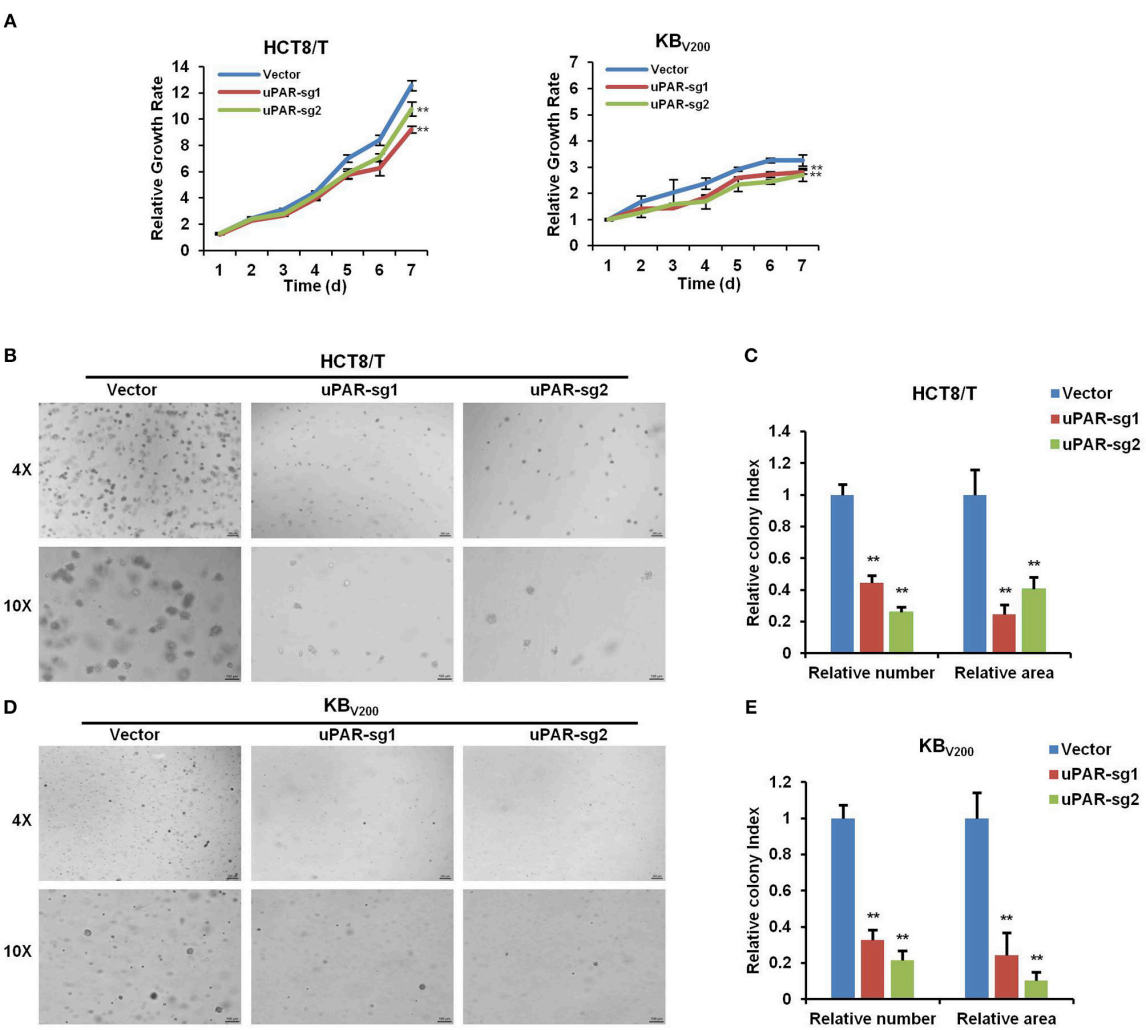
Knockout of uPAR attenuates cell proliferation. **(A)** Cell proliferation was evaluated by MTT assay. Representative spheres images and quantification of HCT8/T **(B,C)** and KB_V200_ **(D,E)** cells were determined by sphere formation assay. ^**^P < 0.01 vs. corresponding control.

To investigate the effect of knockout of uPAR on cell proliferation, we detected cell proliferation by MTT and sphere formation assays. As shown in Figure 3A, knockout of uPAR inhibited the growth of HCT8/T and KB_V200_ cells. Further sphere formation assay showed that knockout of uPAR reduced the sphere number and size of HCT8/T and KB_V200_ cells (Figures 3B–E). These results suggest that knockout of uPAR attenuates cell proliferation.

### Knockout of uPAR Inhibits Cell Migration

**Figure 4:**
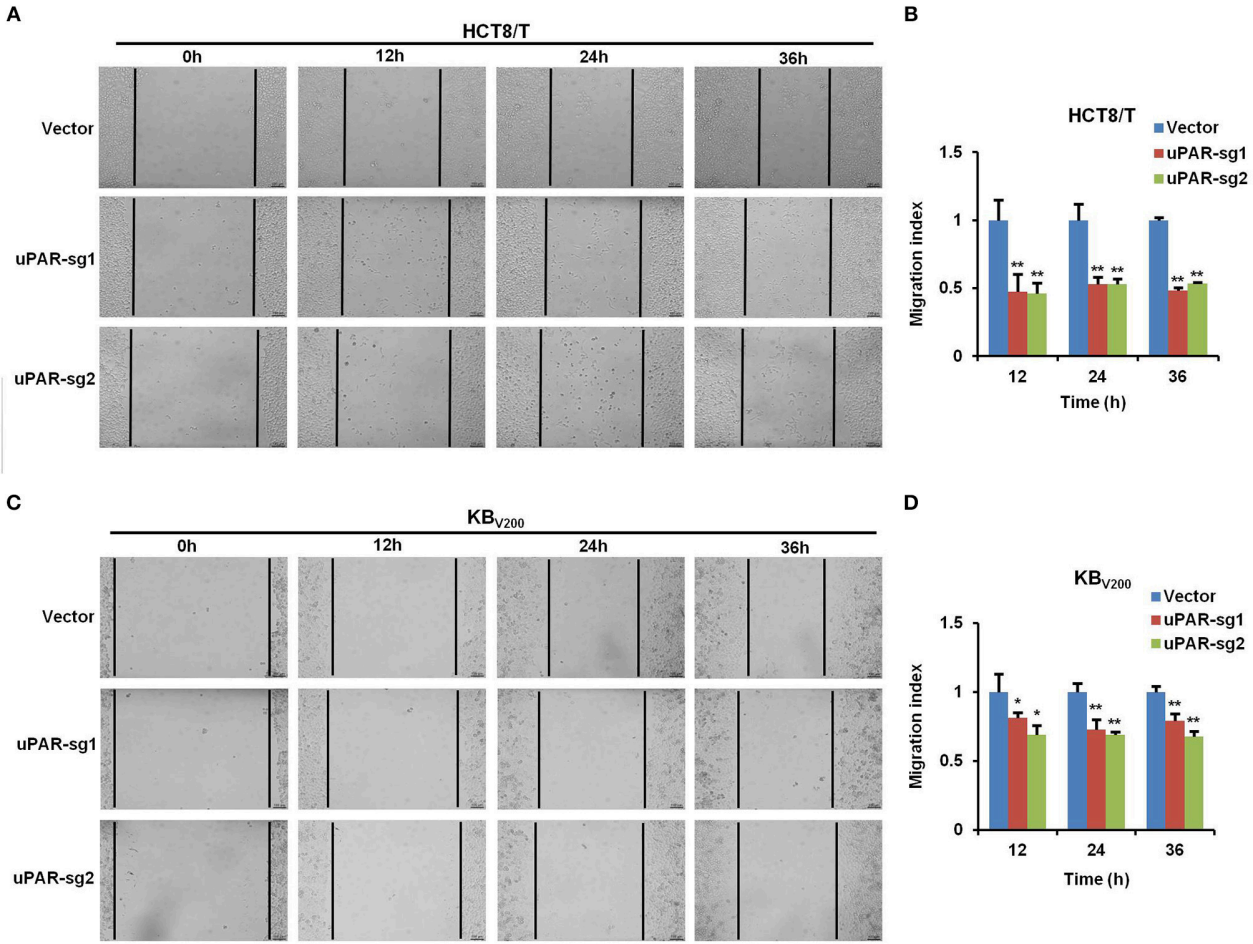
Knockout of uPAR inhibits cell migration. Cell migration was determined with wound healing assay. Representative migration images and quantification of HCT8/T **(A,B)** and KB_V200_ **(C,D)** cells were shown. ^*^P < 0.05 and ^**^P < 0.01 vs. corresponding control.

To examine the effect of knockout of uPAR by CRISPR/Cas9 on cell migration, wound healing assay was used to detect cell migration. The results showed that cell migration was reduced in HCT8/T and KB_V200_ cells with uPAR knockout (Figure 4), indicating that knockout of uPAR inhibits cell migration.

### Knockout of uPAR Inhibits Cell Invasion

**Figure 5:**
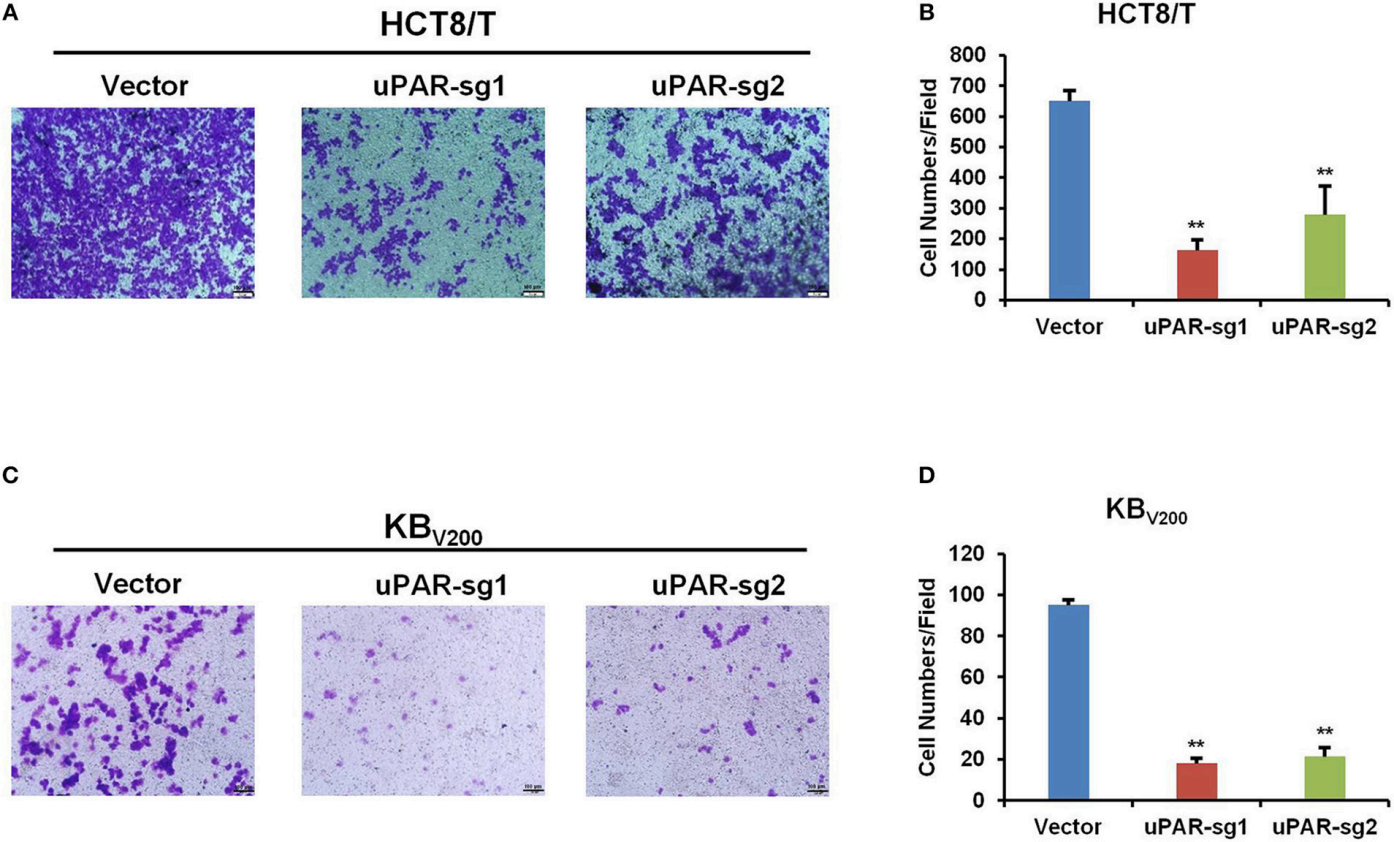
Knockout of uPAR inhibits cell invasion. Cell invasion was determined with transwell assay. Representative invasion images and quantification of HCT8/T **(A,B)** and KB_V200_ **(C,D)** cells were shown. ^**^P < 0.01 vs. corresponding control.

To further evaluate the effect of knockout of uPAR by CRISPR/Cas9 on cell invasion, transwell assay was used to detect cell invasion. As shown in Figure 5, cell invasion was reduced in HCT8/T and KB_V200_ cells with uPAR knockout, suggesting that knockout of uPAR inhibits cell invasion.

### Knockout of uPAR Decreases Multidrug Resistance

**Figure 6:**
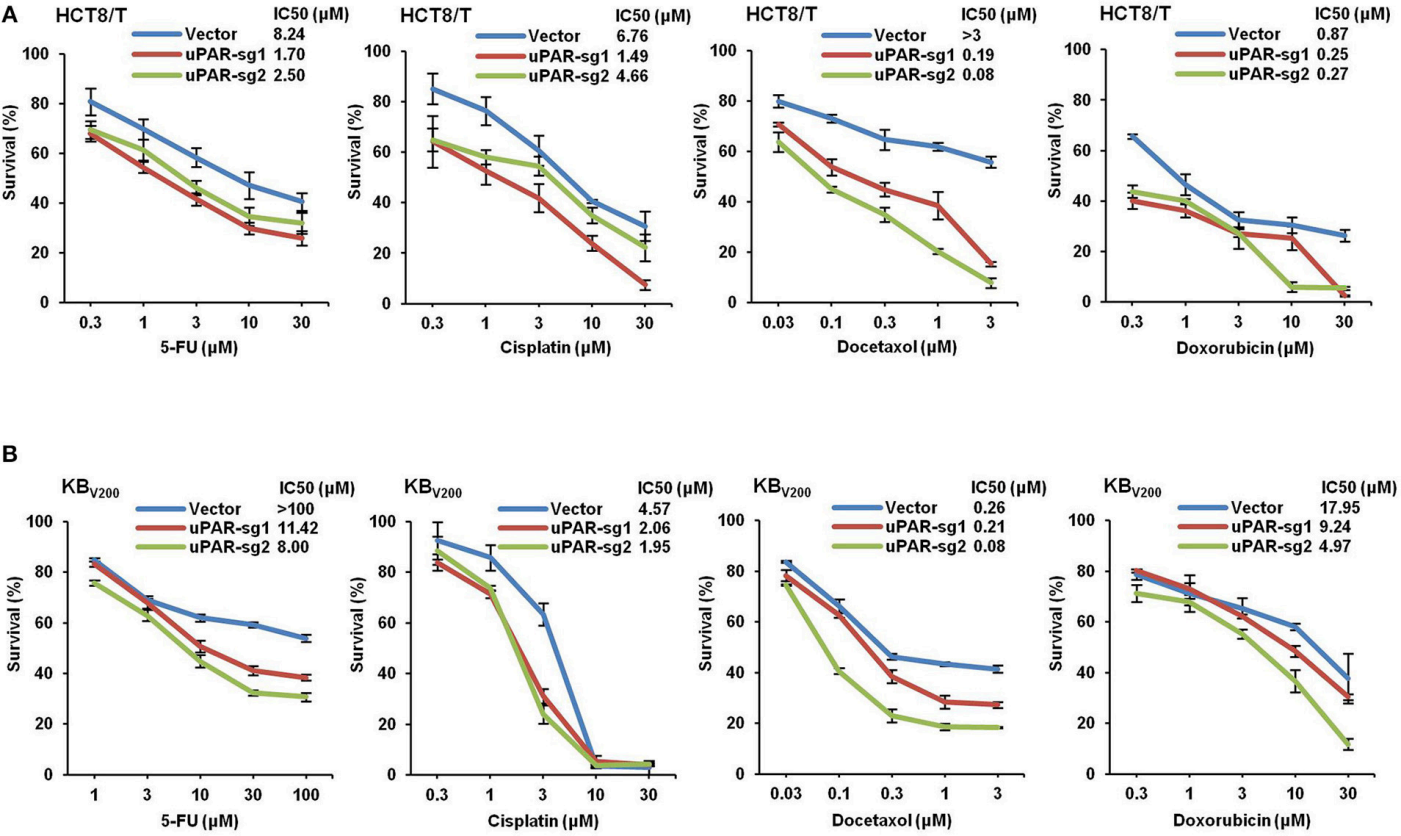
Knockout of uPAR decreases multidrug resistance. Cells survival was measured by MTT assay. The representative growth curve of HCT8/T **(A)** and KB_V200_ **(B)** cells treated with the indicated concentrations of 5-FU, cisplatin, docetaxel, and doxorubicin for 72 h were shown.

To study the effect of knockout of uPAR by CRISPR/Cas9 on multidrug resistance, four chemotherapeutical drugs 5-FU, cisplatin, docetaxel, and doxorubicin were used to treat cells, and cell survival was detected by MTT assays. As shown in Figure 6, the cell survival curves shifted to downward, and IC_50_ values of these four drugs were reduced in HCT8/T and KB_V200_ cells with uPAR knockout. These data indicate that knockout of uPAR suppresses multidrug resistance.

## Discussion

Recently, it has been demonstrated that knockout of uPAR using CRISPR/Cas9 system in mouse neuroblastoma Neuro 2A cells inhibit cell proliferation, reduce the number of Ki-67 positive cells, and down-regulate the mRNA expression level of TrkC receptor (18). In the current study, we successfully targeted uPAR in two cancer cell lines by CRISPR/Cas9 system with two individual sgRNAs. Knockout of uPAR suppresses cell proliferation, migration and invasion. Moreover, knockout of uPAR decreases resistance to 5-FU, cisplatin, docetaxel, and doxorubicin in these cells. Previous studies have shown that high expression of uPAR leads to small cell lung cancer, head and neck squamous cell carcinoma, and malignant pleural mesothelioma resistant to chemotherapy (19–21). uPAR promotes the resistance to tamoxifen in breast cancer by activated ERK1/2 activity (22), and confers the resistance to gefitinib in non-small-cell lung cancer through activated EGFR/pAKT/survivin signal pathway (23). Therefore, uPAR plays important roles not only in cancer malignancy but also in drug resistance.

CRISPR/Cas9 system has been widely applied in exploring the molecular mechanism of tumorigenesis, generating the models for cancer research and identifying the targets for cancer treatment, etc. A genome-wide CRISPR screen shows that loss-of-function mutations of some genes including NF2, PTEN, CDKN2A, TRIM72, FGA, miR-152, miR-345, and so on are able to drive tumor growth and metastasis in a mouse model (24). Using CRISPR/Cas9 technology to target MAN2A1-FER fusion gene inhibits tumor proliferation and metastasis in the mouse models of prostate and liver cancer (25). Colorectal cancer from normal human intestinal epithelium organoids are generated by introducing mutations in the tumor suppressor genes APC, SMAD4 and TP53, and oncogenes KRAS and/or PIK3CA with CRISPR/Cas9 system (26, 27). Liver tumors in mice are occurred by using hydrodynamic injection of CRISPR/Cas9 plasmids and sgRNAs that directly target the tumor suppressor genes PTEN and p53 (28). Mouse pancreatic ductal adenocarcinoma models are established by introducing 13 sgRNAs of different tumor suppressor genes into expression vectors and then transferred them to mouse pancreatic tissue (29). CDC25A is identifies as a determinant of sensitivity to ATR inhibitors by a genome-wide CRISPR screen (30). Deletion of genes such as NF1 and MED12 with CRISPR/Cas9 system is associated with resistance to vemurafenib (31). Moreover, the combination of CRISPR/Cas9 gene editing technology and immunotherapy, especially with CAR-T cell therapy, will have enormous therapeutic potential in leukemia, lymphoma, and some solid tumors (32, 33). Using CRISPR/Cas9 system can produce universal CAR-T cells by simultaneously targeting TCR and HLA-I (34) and enhanced CAR-T cells by deleting T cell inhibitory receptor or signaling molecule genes such as PD1 and CTLA4 (33, 35). We previously have demonstrated that targeting ABCB1 by CRISPR/Cas9-based genome editing reverses ABCB1-mediated multidrug resistance in cancer cells, resulting in the increase of the sensitivity and intracellular accumulation of the anti-cancer drugs (36). Although there are several limitations such as off-targets and delivery in the clinical application of CRISPR/Cas9 technology, it is believed that CRISPR/Cas9 system will benefit cancer patients in the near future.

In summary, our results have demonstrated that targeting uPAR by CRISPR/Cas9-based genome editing causes knockout of uPAR in human cancer cell lines, resulting in attenuation of cell proliferation, migration, invasion and multidrug resistance. Our study offers valuable evidences for the role of uPAR in cancer malignancy and drug resistance.

## Author Contributions

KW, Z-HX, and ZS designed the experiments, performed the experiments, analyzed the data, and wrote the paper. Q-WJ, YY, J-RH, M-LY, M-NW, YL, S-TW, and KL performed the experiments. All authors read and approved the final manuscript.

### Conflict of Interest Statement

The authors declare that the research was conducted in the absence of any commercial or financial relationships that could be construed as a potential conflict of interest.

## References

[1] Mahmood N Mihalcioiu C Rabbani SA. Multifaceted role of the urokinase-type plasminogen activator (uPA) and its receptor (uPAR): diagnostic, prognostic, and therapeutic applications. Front Oncol. (2018) 8:24. 10.3389/fonc.2018.00024 29484286 PMC5816037

[2] Smith HW Marshall CJ. Regulation of cell signalling by uPAR. Nat Rev Mol Cell Biol. (2010) 11:23–36. 10.1038/nrm2821 20027185

[3] Noh H Hong S Huang S. Role of urokinase receptor in tumor progression and development. Theranostics. (2013) 3:487–95. 10.7150/thno.4218 23843896 PMC3706692

[4] Setyono-Han B Sturzebecher J Schmalix WA Muehlenweg B Sieuwerts AM Timmermans M. Suppression of rat breast cancer metastasis and reduction of primary tumour growth by the small synthetic urokinase inhibitor WX-UK1. Thromb Haemost. (2005) 93:779–86. 10.1160/TH04-11-0712 15841327

[5] Zhu M Gokhale VM Szabo L Munoz RM Baek H Bashyam S. Identification of a novel inhibitor of urokinase-type plasminogen activator. Mol Cancer Ther. (2007) 6:1348–56. 10.1158/1535-7163.MCT-06-0520 17431113

[6] Heinemann V Ebert MP Pinter T Bevan P Neville NG Mala C Randomized phase II trial with an uPA inhibitor (WX-671) in patients with locally advanced nonmetastatic pancreatic cancer. J Clin Oncol. (2010) 28:4060 10.1200/jco.2010.28.15_suppl.4060

[7] Andersen LM Wind T Hansen HD Andreasen PA. A cyclic peptidylic inhibitor of murine urokinase-type plasminogen activator: changing species specificity by substitution of a single residue. Biochem J. (2008) 412:447–57. 10.1042/BJ20071646 18318660

[8] Ploug M Ostergaard S Gardsvoll H Kovalski K Holst-Hansen C Holm A. Peptide-derived antagonists of the urokinase receptor. Affinity maturation by combinatorial chemistry, identification of functional epitopes, and inhibitory effect on cancer cell intravasation. Biochemistry. (2001) 40:12157–68. 10.1021/bi010662g 11580291

[9] Luparello C Del Rosso M. *In vitro* anti-proliferative and anti-invasive role of aminoterminal fragment of urokinase-type plasminogen activator on 8701-BC breast cancer cells. Eur J Cancer. (1996) 32A:702–7. 10.1016/0959-8049(95)00657-5 8695276

[10] Mazar AP Ahn RW O'Halloran TV. Development of novel therapeutics targeting the urokinase plasminogen activator receptor (uPAR) and their translation toward the clinic. Curr Pharm Des. (2011) 17:1970–8. 10.2174/138161211796718152 21711234 PMC3188847

[11] Ahmed N Oliva K Wang Y Quinn M Rice G Downregulation of urokinase plasminogen activator receptor expression inhibits Erk signalling with concomitant suppression of invasiveness due to loss of uPAR-beta 1 integrin complex in colon cancer cells. Brit J Cancer. (2003) 89:374–84. 10.1038/sj.bjc.6601098 12865932 PMC2394266

[12] Zhang F Tom CC Kugler MC Ching TT Kreidberg JA Wei Y Distinct ligand binding sites in integrin alpha 3 beta 1 regulate matrix adhesion and cell-cell contact. J Cell Biol. (2003) 163:177–88. 10.1083/jcb.200304065 14557254 PMC2173444

[13] Ghosh S Johnson JJ Sen R Mukhopadhyay S Liu Y Zhang F. Functional relevance of urinary-type plasminogen activator receptor-alpha3beta1 integrin association in proteinase regulatory pathways. J Biol Chem. (2006) 281:13021–9. 10.1074/jbc.M508526200 16510444

[14] Kenny HA Leonhardt P Ladanyi A Yamada SD Montag A Im HK. Targeting the urokinase plasminogen activator receptor inhibits ovarian cancer metastasis. Clin Cancer Res. (2011) 17:459–71. 10.1158/1078-0432.CCR-10-2258 21149615 PMC3073583

[15] Barrangou R Doudna JA. Applications of CRISPR technologies in research and beyond. Nat Biotechnol. (2016) 34:933–41. 10.1038/nbt.3659 27606440

[16] Fellmann C Gowen BG Lin PC Doudna JA Corn JE. Cornerstones of CRISPR-Cas in drug discovery and therapy. Nat Rev Drug Discov. (2017) 16:89–100. 10.1038/nrd.2016.238 28008168 PMC5459481

[17] Yuan ML Li P Xing ZH Di JM Liu H Yang AK. Inhibition of WEE1 suppresses the tumor growth in laryngeal squamous cell carcinoma. Front Pharmacol. (2018) 9:1041. 10.3389/fphar.2018.01041 30323762 PMC6172786

[18] Rysenkova KD Semina EV Karagyaur MN Shmakova AA Dyikanov DT Vasiluev PA. CRISPR/Cas9 nickase mediated targeting of urokinase receptor gene inhibits neuroblastoma cell proliferation. Oncotarget. (2018) 9:29414–30. 10.18632/oncotarget.25647 30034627 PMC6047682

[19] Gutova M Najbauer J Gevorgyan A Metz MZ Weng Y Shih CC. Identification of uPAR-positive chemoresistant cells in small cell lung cancer. PLoS ONE. (2007) 2:e243. 10.1371/journal.pone.0000243 17327908 PMC1800348

[20] Cortes-Dericks L Carboni GL Schmid RA Karoubi G. Putative cancer stem cells in malignant pleural mesothelioma show resistance to cisplatin and pemetrexed. Int J Oncol. (2010) 37:437–44. 10.3892/ijo-0000692 20596671

[21] Huang Z Wang L Wang Y Zhuo Y Li H Chen J. Overexpression of CD147 contributes to the chemoresistance of head and neck squamous cell carcinoma cells. J Oral Pathol Med. (2013) 42:541–6. 10.1111/jop.12046 23413783

[22] Eastman BM Jo M Webb DL Takimoto S Gonias SL. A transformation in the mechanism by which the urokinase receptor signals provides a selection advantage for estrogen receptor-expressing breast cancer cells in the absence of estrogen. Cell Signal. (2012) 24:1847–55. 10.1016/j.cellsig.2012.05.011 22617030 PMC3383391

[23] Zhou J Kwak KJ Wu Z Yang D Li J Chang M. PLAUR confers resistance to gefitinib through EGFR/P-AKT/Survivin signaling pathway. Cell Physiol Biochem. (2018) 47:1909–24. 10.1159/000491071 29961070

[24] Chen S Sanjana NE Zheng K Shalem O Lee K Shi X. Genome-wide CRISPR screen in a mouse model of tumor growth and metastasis. Cell. (2015) 160:1246–60. 10.1016/j.cell.2015.02.038 25748654 PMC4380877

[25] Chen ZH Yu YP Zuo ZH Nelson JB Michalopoulos GK Monga S. Targeting genomic rearrangements in tumor cells through Cas9-mediated insertion of a suicide gene. Nat Biotechnol. (2017) 35:543–50. 10.1038/nbt.3843 28459452 PMC5462845

[26] Matano M Date S Shimokawa M Takano A Fujii M Ohta Y. Modeling colorectal cancer using CRISPR-Cas9-mediated engineering of human intestinal organoids. Nat Med. (2015) 21:256–62. 10.1038/nm.3802 25706875

[27] Drost J van Jaarsveld RH Ponsioen B Zimberlin C van Boxtel R Buijs A. Sequential cancer mutations in cultured human intestinal stem cells. Nature. (2015) 521:43–7. 10.1038/nature14415 25924068

[28] Xue W Chen S Yin H Tammela T Papagiannakopoulos T Joshi NS. CRISPR-mediated direct mutation of cancer genes in the mouse liver. Nature. (2014) 514:380–4. 10.1038/nature13589 25119044 PMC4199937

[29] Maresch R Mueller S Veltkamp C Oellinger R Friedrich M Heid I. Multiplexed pancreatic genome engineering and cancer induction by transfection-based CRISPR/Cas9 delivery in mice. Nat Commun. (2016) 7:10770. 10.1038/ncomms10770 26916719 PMC4773438

[30] Ruiz S Mayor-Ruiz C Lafarga V Murga M Vega-Sendino M Ortega S. A genome-wide CRISPR screen identifies CDC25A as a determinant of sensitivity to ATR inhibitors. Mol Cell. (2016) 62:307–13. 10.1016/j.molcel.2016.03.006 27067599 PMC5029544

[31] Shalem O Sanjana NE Hartenian E Shi X Scott DA Mikkelsen TS. Genome-Scale CRISPR-Cas9 knockout screening in human cells. Science. (2014) 343:84–7. 10.1126/science.1247005 24336571 PMC4089965

[32] Maus MV Grupp SA Porter DL June CH. Antibody-modified T cells: CARs take the front seat for hematologic malignancies. Blood. (2014) 123:2625–35. 10.1182/blood-2013-11-492231 24578504 PMC3999751

[33] Ren JT Zhao YB. Advancing chimeric antigen receptor T cell therapy with CRISPR/Cas9. Protein Cell. (2017) 8:634–43. 10.1007/s13238-017-0410-x 28434148 PMC5563282

[34] Ren JT Liu XJ Fang CY Jiang SG June CH Zhao YB. Multiplex genome editing to generate universal CAR T cells resistant to PD1 inhibition. Clin Cancer Res. (2017) 23:2255–66. 10.1158/1078-0432.CCR-16-1300 27815355 PMC5413401

[35] Hoos A. Development of immuno-oncology drugs - from CTLA4 to PD1 to the next generations. Nat Rev Drug Discov. (2016) 15:235–47. 10.1038/nrd.2015.35 26965203

[36] Yang Y Qiu JG Li Y Di JM Zhang WJ Jiang QW. Targeting ABCB1-mediated tumor multidrug resistance by CRISPR/Cas9-based genome editing. Am J Transl Res. (2016) 8:3986–94. 27725879 PMC5040697

